# Simulation of Preterm Neonatal Brain Metabolism During Functional Neuronal Activation Using a Computational Model

**DOI:** 10.1007/978-1-4939-3023-4_14

**Published:** 2015-06-22

**Authors:** T. Hapuarachchi, F. Scholkmann, M. Caldwell, C. Hagmann, S. Kleiser, A. J. Metz, M. Pastewski, M. Wolf, I. Tachtsidis

**Affiliations:** 1 CoMPLEX, University College London, London, UK; 2 Department of Medical Physics and Bioengineering, University College London, London, UK; 3 Division of Neonatology, Biomedical Optics Research Laboratory, University Hospital Zurich, Zurich, Switzerland; 4 Clinic of Neonatology, University Hospital Zurich, Zurich, Switzerland

**Keywords:** Mathematical model, fNIRS, Haemodynamics, Autoregulation, Stimulus – evoked functional response

## Abstract

We present a computational model of metabolism in the preterm neonatal brain. The model has the capacity to mimic haemodynamic and metabolic changes during functional activation and simulate functional near-infrared spectroscopy (fNIRS) data. As an initial test of the model’s efficacy, we simulate data obtained from published studies investigating functional activity in preterm neonates. In addition we simulated recently collected data from preterm neonates during visual activation. The model is well able to predict the haemodynamic and metabolic changes from these observations. In particular, we found that changes in cerebral blood flow and blood pressure may account for the observed variability of the magnitude and sign of stimulus-evoked haemodynamic changes reported in preterm infants.

## Introduction

Our research focuses on the development of a family of computational models of cerebral metabolism, primarily to investigate the effects of stimuli and physiological insults, and to inform the clinical treatment of brain brain injury. This work has so far centred on human adult [1] and piglet cerebral activity [2]. We have recently extended our focus to the preterm neonatal brain.

A number of studies investigating functional activity in neonates using functional near infrared spectroscopy (fNIRS) have observed different haemodynamic responses. Inconsistent results have been reported in literature regarding the characteristics of stimulus-evoked changes (i.e. magnitude and sign) in oxyhaemoglobin (HbO_2_) and deoxyhaemoglobin (HHb). In particular, some studies report a decrease in HHb (an adult-like response) while others report the opposite. In order to research the mechanisms of these responses we have adapted an existing model of adult cerebral metabolism (BrainSignals) [1] to the preterm neonatal brain. In this paper, we (1) present a model of metabolism and haemodynamics in the preterm brain, (2) use the model to simulate observations of two published preterm functional response studies and (3) use the model to predict recently collected data from a stimulus-evoked haemodynamic response study in preterm neonates.

## Modelling Functional Activation in the Developing Preterm Brain

**Fig. 14.1:**
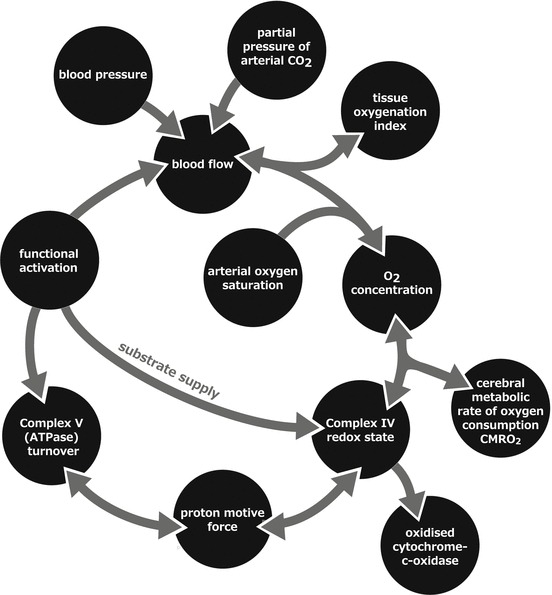
A simple schematic of the model. Model inputs are blood pressure, arterial oxygenation saturation, partial pressure of arterial carbon dioxide and functional activation

**Fig. 14.2:**
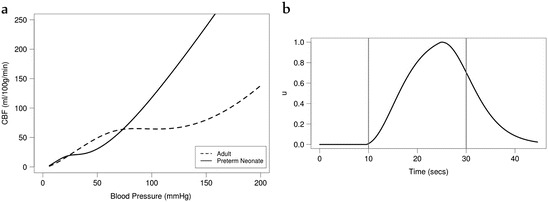
(**a**) Autoregulation curve—cerebral blood flow (CBF) against blood pressure—for the adult BrainSignals model and the preterm neonate model. (**b**) Demand as a model input, using a haemodynamic response function, to simulate functional activation

The original BrainSignals model simulates blood circulation and energy metabolism. It uses a combination of differential equations and algebraic relations to mimic biochemical reactions and processes in a brain cell and the immediate vasculature. The model predicts in particular responses to changes in arterial blood pressure, oxygenation, carbon dioxide levels and functional activation. Figure 14.1 illustrates a simple schematic of the model. This model was adapted to the human neonate by altering a number of physiological parameters known to be significantly different in the young (a method similar to that employed in developing the piglet model [2]). These parameters are listed in Table 14.1. In particular, the reduction of normal arterial blood pressure (BP) was seen to have a significant effect on the behaviour of the model. Figure 14.2a shows the autoregulation curve of the adult model and the neonatal model for preterm neonates, comparable to approximations found in literature [11]. In order to simulate functional activation, the model uses a dimensionless parameter *u* which represents demand. A change in *u* produces a response in vascular smooth muscle and affects ATP production by influencing the driving force for complex V in mitochondria.

**Table 14.1:** BrainSignals parameters modified to represent the preterm neonatal brain

Parameter	Description	Units	BrainSignals	Preterm neonate	Source
CBF_n_	Normal cerebral blood flow (CBF)	ml 100 g^−1^ min ^−1^	49	19.8	[3]
[CCO]_tis_	Total concentration of cytochrome-c-oxidase (CCO) in tissue	μM	5.5	2.2	[4, 5]
Cu_A,frac,n_	Normal fraction of oxidised CCO		0.8	0.67	[5]
CMRO_2,n_	Normal cerebral metabolic rate of oxygen consumption (CMRO_2_)	μmol 100 g^−1^ min ^−1^	155	40.865	[6]
P_a_ and P_a,n_	Mean arterial blood pressure	mmHg	100	30	[7]
[Hbtot] and [Hbtot]_n_	Total concentration of haemoglobin in blood	mM	9.1	9.75	[8]
					[7]
V_blood,n_	Normal brain blood fraction		0.04	0.0233	[9]
P_ic_ and P_ic,n_	Intracranial pressure	mmHg	9.5	5.1	[10]

## Model Simulations

**Fig. 14.3:**
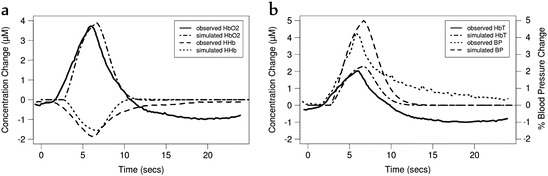
Model simulated and observed haemodynamic response of the Kozberg et al. study [12], investigating functional response in rats, with an increase in demand and blood pressure (BP). (**a**) Changes in deoxy- and oxy- haemoglobin (HHb, HbO2) concentrations. (**b**) Changes in BP and total haemoglobin (HbT)

**Fig. 14.4:**
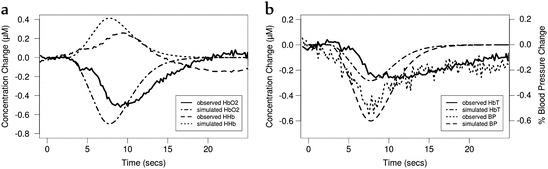
Model simulated and observed haemodynamic response of the Kozberg et al. study [12], investigating functional response in rats, with a slight decrease in arterial radius and blood pressure (BP). (**a**) Changes in deoxy- and oxy- haemoglobin (HHb, HbO2) concentrations. (**b**) Changes in BP and total haemoglobin (HbT)

Kozberg et al. [12] conducted a study in postnatal rats age-equivalent to human newborns. Although our model is focused on the human neonate, we were interested in replicating the qualitative response observed in the animals as they were subjected to a somatosensory stimulus. The rats which exhibited a rise in BP in response to the stimulus also showed an increase in HbO_2_ and total haemoglobin (HbT) and a decrease in HHb, where the increase in HbO_2_ was greater than the decrease in HHb (functional hyperemia). However, some rats showed a slight decrease or no change in BP. In these animals, the opposite results were observed—a decrease in HbO_2_ and HbT and a rise in HHb. These conflicting results were attributed to a lack of functional hyperemia and an overarching effect of arterial vasoconstriction in the latter group. In order to model these results, we increased the model’s demand parameter *u* to simulate functional activation with the shape of a steep rising haemodynamic response function (HRF). The demand was calculated as *u* = *1.0* + *α HRF* where *α* is a real number (Fig. 14.2b). We simulated BP also using the HRF (*P*_*a*_ = *P*_*a*,*n*_ − *β HRF*). The values for *α* and *β* were optimised to achieve the best fit of our model simulations to the observed results. Arterial oxygen saturation (SaO_2_) and partial pressure of carbon dioxide (PaCO_2_) were assumed to remain constant. The model is able to predict changes in haemodynamics (ΔHbO_2_, ΔHHb, ΔHbT) which we compare against experimental data. The model is also capable of simulating cerebral blood flow (CBF) and the cerebral metabolic rate of oxygen consumption (CMRO_2_). For the first group of animals who showed an increase in blood pressure, we used *α* = 2 and *β* = 1.5. Although we attempted to include vasoconstriction as observed in the study, we found that preventing the dilation of the vessels reversed our results. For the second group we reduced the arterial radius by 1 % (*r* = *r*_*n*_ (*1* − *0.01 HRF*)) and used *β* = 0.18. We did not need to increase the demand *u*, suggesting that an increase in oxygen consumption was not required to produce this response. Figures 14.3 and 14.4 illustrate these results, which are very closely comparable to the original data obtained by Kozberg et al. [12].

**Fig. 14.5:**
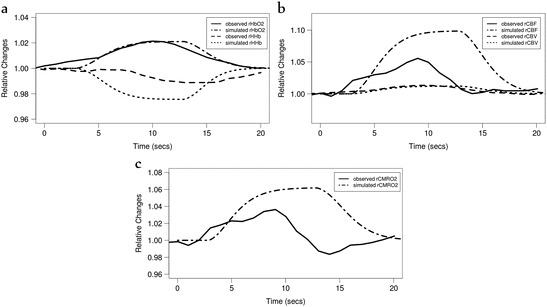
Model simulated and observed haemodynamic response of the Roche-Labarbe et al. study [13], investigating functional response in human preterm neonates, with an increase in demand. (**a**) Relative changes in oxy- and deoxy- haemoglobin (rHbO2, rHHb), (**b**) cerebral blood volume (rCBV) and cerebral blood flow (rCBF) and (**c**) cerebral metabolic rate of oxygen consumption (rCMRO2)

The second study we investigated was conducted in preterm human neonates by Roche-Labarbe et al. [13]. They observed a decrease in HHb and an increase in HbO_2_, CBF, CMRO_2_ and cerebral blood volume (CBV) in response to a somatosensory stimulus. We were able to replicate these results relatively well by simply increasing the model’s demand (α = 0.5) (Fig. 14.5). However, our predicted CBF and CMRO_2_ was higher and HHb slightly lower than that observed.

We also simulated data from a functional near infrared spectroscopy (fNIRS) study conducted at University Hospital Zurich (USZ) which investigates the functional response in the preterm brain evoked by a visual stimulus. A blinking pocket LCD display was used as the stimulus and changes in tissue oxygenation and haemodynamics were measured over the prefrontal cortex using a novel spatially-resolved NIRS device (OxyPrem). Measurements of changes in HbO_2_, HHb and HbT were averaged over repeated stimuli. Characteristics of two preterm subjects are detailed in Table 14.2.

**Table 14.2:** Physiological characteristics of the two preterm infant subjects

	Gestational age (weeks)	Actual age (weeks)	Weight (g)	Haematocrit (%)	Haemoglobin (g/dL)	FiO2 (%)	Baseline SpO2 (%)	Baseline heart rate (BPM)
Neonate 1	33.3	33.4	2380	52.5	17.08	21.0	95	148
Neonate 2	25.9	39.0	3460	30.0	9.70	28.0	92–95	165

**Fig. 14.6:**
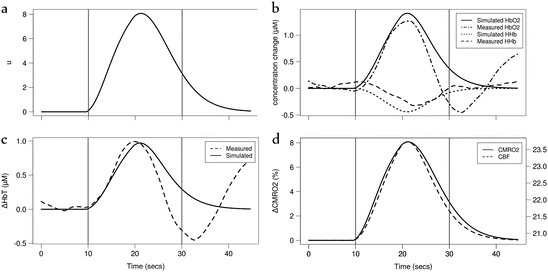
Model simulation of Neonate 1 of the USZ study. Simulated haemodynamic response with (**a**) an increase in demand *u. Vertical lines* indicate stimulus period. Measured and simulated changes in (**b**) oxy- and deoxy- haemoglobin (ΔHbO2, ΔHHb) and (**c**) total haemoglobin (ΔHbT). (**d**) Simulated cerebral metabolic rate of oxygen consumption (CMRO2) cerebral blood flow (CBF)

We assumed that the SaO_2_, PaCO_2_ and BP remain constant in the first instance. In Neonate 1, the measurements showed an increase in ΔHbO_2_ and ΔHbT during the stimulus, and a decrease in ΔHHb (Fig. 14.6). We were able to simulate this response in our model by a simple increase in demand (α = 0.7). Modelled CBF and CMRO_2_ showed a corresponding rise during the stimulus.

**Fig. 14.7:**
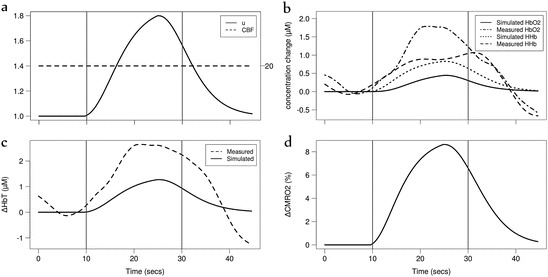
Model simulation of Neonate 2 of the USZ study. Simulated haemodynamic response with (**a**) an increase in demand u and CBF maintained constant. *Vertical lines* indicate stimulus period. Measured and simulated changes in (**b**) oxy- and deoxy- haemoglobin (ΔHbO2, ΔHHb) and (**c**) total haemoglobin (ΔHbT). (**d**) Simulated cerebral metabolic rate of oxygen consumption (CMRO2)

In Neonate 2, a similar increase in CMRO_2_, ΔHbO_2_ and ΔHbT was observed during the stimulus. However, an increase in ΔHHb was also observed (Fig. 14.7). We were able to simulate this response in ΔHHb by an increase in demand (α = 0.7) while maintaining CBF constant at its normal value throughout the stimulus. We were able to better predict ΔHbO_2_ and ΔHbT by adding a decrease in blood pressure during the stimulus (*P*_*a*_ = *P*_*a*,*n*_ − *β HRF* with *β* = 7) to match the magnitude of ΔHbO_2_ and ΔHbT. However this decrease was too large to be physiologically likely within this timeframe (−7 mmHg).

## Discussion

The autoregulatory capacity of the preterm neonatal brain remains unclear. However, as our model simulation (Fig. 14.2a) suggests, preterm neonates may be able to maintain constant blood flow only within a very narrow range of blood pressure values. Studies have shown that the response of HHb to a functional stimulus is sometimes ‘inverted’ in preterm neonates as compared to adults. Our efforts to simulate the haemodynamic responses observed in studies performed by Kozberg et al. [12] and Roche-Labarbe et al. [13] show that the preterm model is capable of simulating the varied functional responses observed. We observed that the model predicted an HHb decrease in response to the stimulus unless we imposed vasoconstriction (as observed by Kozberg et al. [12]). Decreasing the radius of the blood vessel resulted in the ‘inverted’ response. The model was also able to simulate fNIRS data from the USZ study relatively well. In Neonate 1 we observed a similar response of hyperemia as observed in the Roche-Labarbe et al. study. In Neonate 2, the observed rise in ΔHHb was simulated here by a constant CBF (Fig. 14.7). However, the magnitude of ΔHbO_2_ and ΔHbT response was not sufficiently simulated. Neonates 1 and 2 are markedly different in both gestational and actual age (Table 14.2). The former, being older, is more likely to have a developed autoregulatory capacity although we note that both subjects showed an increased HbT response. Their differences in haematocrit and haemoglobin are also notable. Indeed it has been suggested previously that HbT may have an effect on the haemodynamic response in newborns [14]. However, by changing baseline HbT, we did not observe an effect on the magnitude or shape of the model’s simulations.

In adapting the model to the human neonate we did not alter the normal radius of the blood vessel and other similar parameters, such as the thickness and muscular tension of the vessel wall. Our current work suggests that these baseline values do not have a significant effect on the results. However, these changes will be made in a forthcoming version of the model.

We aim to further investigate the functional response in neonates using our model. Our initial results suggest that the interaction of many variables affect this response including CBF, BP and the varied stages of development. This makes it very difficult to define a ‘normal’ functional response for all neonates.
